# Investigation of Wheat Germ and Oil Characteristics with Regard to Different Stabilization Techniques

**DOI:** 10.17113/ftb.58.03.20.6638

**Published:** 2020-09

**Authors:** Derya Arslan, M. Kürşat Demir, Ayşenur Acar, Fatma Nur Arslan

**Affiliations:** 1 Department of Food Engineering, Faculty of Engineering and Architecture, Necmettin Erbakan University, Koycegiz Campus, Konya, Turkey; 2 Department of Chemistry, Faculty of Science, Karamanoğlu Mehmetbey University, Karaman, Turkey; 3 Van’t Hoff Institute for Molecular Sciences, Analytical Chemistry Group, University of Amsterdam, Science Park 904, 1098 XH, Amsterdam, Netherlands

**Keywords:** wheat germ oil, oil stabilization, lipoxygenase, tocopherols, tocotrienols

## Abstract

**Research background:**

Utilization of wheat germ and wheat germ oil is limited due to high enzymatic activity and the presence of unsaturated fatty acids, which require stabilization techniques to overcome this problem.

**Experimental approach:**

In this study, the effects of stabilization methods (dry convective oven heating at 90 and 160 °C, microwave radiation at 180 and 360 W, and autoclave steaming) on both wheat germ and its oil were evaluated.

**Results and conclusions:**

Steaming caused the most dramatic changes in lipoxygenase activity, free fatty acid content, DPPH radical scavenging activity, and mass fractions of tocopherols and tocotrienols. Lower peroxide values were measured in the oil samples treated with convectional heating (160 °C) and steaming at temperatures above 100 °C. However, *p*-anisidine values of samples treated at higher temperatures were considerably greater than those of samples stabilized at lower temperatures. Oven heating at 160 °C was also one of the most effective treatments, after steaming, for the inactivation of lipoxygenase. Steaming significantly reduced mass fraction of total tocopherols, which was directly associated with the greater loss of β-tocopherol content. On the contrary, γ- and δ-tocopherol and tocotrienol homologues were abundant with higher amounts in steamed samples. α-Tocopherol and γ-tocotrienol were the most resistant isomers to stabilization processes.

**Novelty and scientific contribution:**

This study shows that the high temperature oven heating method, which is widely used in the industry for thermal stabilization of wheat germ, does not provide an advantage in oxidative stability compared to steaming and microwave applications. Steaming delayed oxidation in the germ, while further inhibiting lipoxygenase activity. Moreover, tocotrienols were more conservable. In industrial application, low-power microwave (180 instead of 360 W) and oven heating at lower temperature (90 instead of 160 °C) would be preferable.

## INTRODUCTION

Wheat germ is a highly nutritional product, which contains about 10–15% lipids, 26–35% proteins, 17% sugars, 1.5–4.5% fibre and 4% minerals (*1*). Germ contains approx. 11% oil, also with considerable amount of bioactive compounds (*2*). Wheat germ oil is the one with the highest tocopherol content among vegetable oils. In addition, the ratio of polyunsaturated fatty acids, mainly linoleic and linolenic acids, is quite high (almost 80% of the oil) (*3*). Despite its superior health benefits, wheat germ is mostly used as animal feed and rarely for human nutrition (*4*, *5*). The germ is used in bread, snacks and cereals in the field of food technology. In addition, its oil has an important place in the food, medicine and cosmetics industry (*6*). The mechanical processes applied during wheat milling cause the cells to break down and the intracellular oil becomes more prone to oxidation. Degradation occurs by the action of oxidative and hydrolytic enzymes such as lipase and lipoxygenase (LOX) on unsaturated fatty acids (*1*). Therefore, lipase and LOX inactivation is required to prolong the storage stability of the wheat germ. At the same time, due to the high nutrient content, microorganisms cause the wheat germ to agglomerate, ferment or develop mould.

For limiting the wheat germ enzymatic activity, several stabilization processes have been applied: extrusion (*7*), microwave heating (*8*), infrared radiation treatment (*9*), steaming (*10*), dehydration (*11*), atmospheric cold plasma (*12*), as well as chemical preservation, *e.g.* addition of antioxidants (*13*) or alkalis (*14*). Currently, the most commonly used heat treatment methods to prevent oxidation are dry heating and steaming (*15*).

The aim of this study is to evaluate the effects of different thermal treatments on the bioactive components and oxidative stability of wheat germ and its oil, with special emphasis on the individual tocopherol and tocotrienol isomers. Both wet and dry treatments of wheat germ samples and their corresponding oil were investigated to determine the relationship, if any, between the enzymatic activity, oxidative stability, non-saponifiable components and some physical characteristics.

## MATERIALS AND METHODS

### Materials

Fresh wheat germ was obtained from the variety *Tiriticum aestivum* L. just after the milling at a local flour mill owned by the ONEL Company, Konya province, Turkey. A sufficient amount of wheat germ was used for oil extraction and the remaining samples were stored in air tight packages at -18 °C for enzyme analysis. All reagents and solvents used were of analytical grade.

### Wheat germ treatment and extraction of oil

**Fig. 1:**
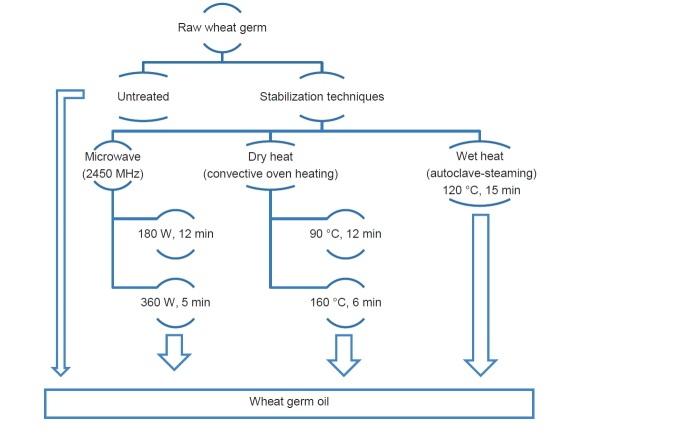
Schematic presentation of experimental process

A mass of 1 kg of wheat germ was weighed, in duplicate, and treated as follows: dry heat (oven), wet heat (autoclave, steaming) and microwaves (Fig. 1). Oven drying was performed at 90 and 160 °C for 12 and 6 min, respectively, using a laboratory type electric oven equipped with a forced hot-air circulation system (KD 200; Nüve, Ankara, Turkey). The samples were equally spread onto trays (42 cm×33 cm×3 cm (length×width×thickness)) to the approximate thickness of 1.0 cm. The samples were placed in the oven after the set temperature was reached. The samples were treated for 12 and 5 min at 180 and 360 W (2450 MHz), respectively, in a commercial microwave oven (SolarDOM™, LG, Seoul, South Korea) using its turntable (35 cm diameter, sample thickness approx. 1.0 cm). Wet heat treatment was carried out on samples weighed in heat-resistant screw capped glass jars in an autoclave (WAC-60; WiseClave, Seoul, South Korea) set at 121 °C and 15 min. At the end of the treatments, samples were cooled to room temperature in a desiccator. Each sample was put in a separate polyethylene plastic bag and stored at -18 °C. An untreated sample was used as control.

A mass of 300 g of each stabilized wheat germ sample were extracted with hexane (Merck, Darmstadt, Germany) at ratio 1:10 (*m*/*V*) and vibrated on shaking water bath (WSB-30; WiseBath, Seoul, South Korea) at 30 °C for 2 h. The extract was filtered through No. 1 filter paper (Whatman International Ltd., Maidstone, UK) in a Büchner funnel. The extraction procedures were repeated twice under the same conditions. Solvent was removed at 40 °C using a rotary evaporator (Laborota 4000; Heidolph Schwabach, Germany). The obtained oil was kept in a glass container at 4 °C until further analysis.

### Analytical determinations of wheat germ oil

Free fatty acids and peroxide value (PV) were determined according to AOCS official method Ca 5a-40 (*16*). *p*-Anisidine value (*p*AV) was determined by using the AOCS official method Cd 18-90 (*17*). Total oxidation (TOTOX) value was calculated with the following equation (*18*):

TOTOX=2PV+*p*AV /1/

### Rancimat analysis

Rancimat values were expressed as the oxidation induction time (h), using the Rancimat apparatus, model 892 (Metrohm Co., Herisau, Switzerland). A mass of 3 g of oil sample heated at 120 °C was aerated with the air flow 20 L/h. The induction time was expressed in hours. The rancimat measurements were carried out in triplicate (*19*).

### Determination of tocopherols and tocotrienols

The stock standard solutions of tocopherol and tocotrienol isomers in hexane (Merck) were prepared at a concentration of 1000 µg/mL. The linear calibration curve (10-500 μg/mL) was prepared by dilution with hexane stock standard solution. A volume of 1 mL hexane was added to oil samples ((1.00±0.01) g) in glass tubes which were shaken for 2 min. The samples were filtered through 0.45-µm polytetrafluoroethylene membrane filters before injection. Analyses of tocopherols and tocotrienols were performed on an Agilent 1260 Infinity II system (Agilent Technologies Inc, Wilmington, DE, USA) equipped with a model G7121A fluorescence detector (FLD). Data were recorded using Agilent’s Chemstation B.03.02-2008 data processor. The separations were performed on a Develosil C_30_ (250 mm×4.6 mm, 5 µm; Phenomenex Inc., Torrance, CA, USA) stainless-steel column. Mixture of solvent A (methanol/water 91:9) and solvent B (methyl *tert*-butyl ether/methanol/water 80:18:2) (Sigma-Aldrich, Merck, Taufkirchen, Germany) in gradient program, different flow rates and temperatures were used to perform optimum separations. The mobile phase solvents A and B reported by Knecht *et al.* (*20*) were used with a modified gradient program. The following gradient program of solvents A and B was applied: 0–20.5 min 0% B, 20.5–25 min 0–40% B, 25–36 min 40% B, 36–46 min 40–55% B, 46–48 min 55–80% B, 48–51 min 80% B, 51–53 min 80–0% B, 53–63 min 0% B. The flow rate was 0.5 mL/min, and temperature was set at 5 °C. A volume of 5 µL of the sample was injected into the system. Fluorescence detector was set at wavelengths of 296 and 330 nm.

### Non-enzymatic browning index

The oil samples were diluted with chloroform (SigmaAldrich, Merck, Steinheim, Germany) at the ratio 1:20 (*m/V*). The absorbance of the solution was measured spectrophotometrically (Libra S21-S22; Biochrom Ltd, Cambridge, UK) at 420 nm to represent the browning Index (*21*).

### DPPH radical scavenging activity

The oil samples of 0.2 g were mixed with 900 μM DPPH (1,1-diphenyl-2- picrylhydrazyl; Fluka, Steinheim, Germany) in toluene (ISOLAB, Wertheim, Germany) and the final concentration was made to 750 μM. The mixture was incubated for 20 min at room temperature ((24±1) °C) and the absorbance was measured at 515 nm against blank sample. The inhibitory percentage of DPPH was calculated using the following equation (*22*):

Inhibition=(*A*_blank_−*A*_sample_/*A*_blank_)·100 /2/

### Lipase activity assay

The reaction solution was prepared with a mixture of 100 mM phosphate buffer (pH=7.0), ethanol and 50 μM *p*-nitrophenol butyrate (*p*NPB) (all these chemicals were from Merck) at 95:4:1 ratio, respectively. A volume of 2.7 mL of the reaction solution was added to 0.3 g wheat germ, and the reaction continued at 60 °C for 15 min. The reaction tubes were allowed to stand at -18 °C for 8 min and the reaction was stopped. The absorbance at 400 nm was measured (*23*). One unit (U/mg) of lipase was defined as the amount of enzyme required for the formation of 1 μmol *p*-nitrophenol per min at 400 nm, 60 °C and pH=7.0. Lipase activity was calculated as follows:

Specific activity= (Δ*A*·*V*_f_)/(*ε*_400 nm_·*t*·*b*·*V*_e_) /3/

where *A* is the absorbance, Δ*A* is the difference between the *A*_400 nm_ of the sample and *A*_400 nm_ of the blank, *V*_f_ is total reaction volume (2.7 mL), *ε*_400 nm_ is molar absorption coefficient of *p*-nitrophenol (5.081 mM^-1^cm^-1^), *t* is reaction time (15 min), *b* light path (1 cm) and *V*_e_ enzyme mass in reaction mixture (0.3 g).

### Lipoxygenase activity

Sample solution was prepared by mixing 10 g wheat germ sample with 100 mL of 0.1 M acetic acid buffer (pH=4.7) for 30 min, followed by centrifugation (Heraeus Multifuge X3R; Thermo Fisher Scientific, Basingstoke, UK) at 12 298×*g* for 10 min. The substrate solution was prepared by mixing 157.2 µL pure linoleic acid, 157.2 µL Tween 20 (Sigma-Aldrich, Merck, Taufkirchen) and 10 mL deionized distilled water. The solution was clarified by adding 1 mL of 1 M sodium hydroxide (Merck). Then it was diluted to 200 mL with 0.2 M sodium phosphate buffer, pH=7.0; which gave a 2.5 mM final concentration of linoleic acid (Fluka Sigma-Aldrich, St. Louis, MO, USA). The substrate solution was flushed with oxygen gas for at least 2 min to give an initial absorbance at 234 nm of 0.3-0.4, and allowed to equilibrate in a water bath at 25 °C. The total reaction volume was 3 mL, which contained 2.7 mL substrate solution and 0.3 mL sample solution. One unit of enzyme activity was defined as an increase in the absorbance at 234 nm for 0.001/min under assay conditions (*24*). The molar absorption coefficient for the conjugated diene of linoleic acid was 23 000 M^-1^cm^-1^ (*25*).

### Statistical analysis

Variance analysis was performed with SPSS statistical software v. 17.0 (*26*). Duncan’s multiple range test was used to assess significant differences between the treatments (p*<*0.05). All the determinations were made in triplicates.

## RESULTS AND DISCUSSION

### Influence of stabilization methods on FFA content of wheat germ oil

FFA content is a typical indicator of the degree of hydrolytic rancidity of lipids. The FFA content expressed as mass fraction of oleic acid of raw germ samples ranged from 2.7 to 5.5% (Table 1). The untreated samples had significantly higher FFA content than the oil in stabilised samples. There was no significant difference in the FFA content between thermally treated samples. Steaming increased the reduction in FFAs.

**Table 1:** Some analytical parameters and oxidative stability of wheat germ oil

Parameter	Untreated	*P*(microwave)/W		*t*(dry heat)/° (convective oven heating)		Wet heat (steaming)
360	180	90	160
Free fatty acids (as *w*(oleic acid)/%)	(5.4±0.8)^a^	(3.1±0.4)^b^	(3.9±0.3)^b^	(3.4±0.5)^b^	(3.5±0.2)^b^	(2.7±0.1)^b^
Peroxide value/(mmol/kg)	(6.8±0.1)^a^	(4.7±0.4)^c^	(5.6±0.4)^b^	(5.7±0.2)^b^	(2.400.5)^d^	(2.6±0.8)^d^
*p*-anisidine (pAV)	(3.38±0.05)^d^	(4.2±0.1)^c^	(1.16±0.02)^e^	(4.19±0.06)^c^	(7.3±0.13^a^	(5.86±0.07)^b^
TOTOX/(mmol/kg)	(33.9±9.4)^a^	(25.2±4.7)^ab^	(26.6±11.8)^ab^	(30.2±11.3)^ab^	(18.1±0.8)^b^	(17.7±0.1)^b^
Induction time/h	(2.54±0.08)^a^	(1.7±0.3)^ab^	(1.8±0.2)^ab^	(2.0±0.9)^ab^	(1.5±0.5)^b^	(1.9±0.2)^ab^
NEBI	(0.47 ±0.05)	(0.39 ±0.05)	(0.43±0.06)	(0.48 ±0.03)	(0.49 ±0.07)	(0.45 ±0.03)
DPPH inhibition/%	(58.2±0.3)^b^	(54.6±1.8)^c^	(64.9±1.8)^a^	(58.3±1.9)^b^	(58.0±1.5)^b^	(48.9±1.6)^d^

Results are expressed as mean value±standard deviation of three determinations. Different lower-case letters in the same row represent significant differences at p<0.05 (comparison between stabilized and unstabilised samples). NEBI=non-enzymatic browning index

In the study of Hu *et al.* (*27*), superheated steam and conventional hot air treatments did not affect the FFAs of wheat bran. Their explanation was that hydrolytic rancidity does not happen during stabilization treatments. Similar findings have also been reported for brown rice stabilized by dry heat, steam and microwaves, where the reduction in FFA of stabilized samples was attributed to the inhibitory effect of stabilization on the lipase (*28*). According to our results, wet heat stabilization was more effective in lowering the FFA values (2.7%) than the other stabilization methods applied (3.1-3.9%), even though the difference was not statistically significant. Bergonio *et al.* (*28*) also reported that microwave and steaming were more effective in reducing acidity. This phenomenon was explained by the higher resistance of proteins to denaturation in a dry environment than in a wet environment (*29*).

### Peroxide, p-anisidine and TOTOX values of wheat germ oil

Germ oil samples showed peroxide value (PV) expressed as oxygen equivalents per kg of oil from 5.4±0.6 to 15.3±0.5 mmol/kg, which did not exceed the legal limits (15 mmol/kg) in all samples according to FAO/WHO (*30*) (Table 1). The stabilization resulted in lower PV (between 5.4 and 12.9 mmol/kg, as raw wheat germ had PV of 15.2 mmol/kgl. The lowest PVs were measured in the oil after convectional heating at 160 °C (5.4 mmol/kg) and steaming (5.9 mmol/kg) at above 100 °C. These were followed by high power microwave-treated samples (10.5 mmol/kg), but with almost twice the values of dry convectional heating (160 °C) and steaming. The longer exposure of wheat germ tohumidity inside the chamber during low temperature microwave treatment could be the reason for higher PV of the samples. These results show that output power is more effective than treatment time in the stabilization by microwave radiation.

During treatments at above 100 °C, such as steaming and convectional dry heating, the inactivation of the enzymes was much higher and thus peroxide formation was slower. In microwave treatments, lower values were obtained in the high-power process. Therefore, it can be emphasized that the applied output power is more effective than time in microwave radiation applications.

According to the results of Li *et al.* (*31*), raw germ showed higher PV than stabilized samples; however, long time (45 and 60 min) and temperature exposure to short-wave infrared radiation were not effective. Hu *et al.* (*27*) reported that superheated steam process avoided hydroperoxide formation in wheat bran, while hot air stabilization increased PVs. The lack of or brief contact with oxygen is shown as the underlying cause of lower peroxide development in steam-heated samples (*27*). The overall preventive effect of thermal stabilization against oxidation was ascribed to the high tocopherol content in wheat germ oil since the lipid radicals generated during the treatments were stabilised by these compounds.

As for the results of the *p*-anisidine value (*p*AV), which give an idea about the secondary oxidation products of lipids, mainly aldehydes such as 2,4-dienals and 2-alkenals, all of the stabilization processes except microwave (180 W) significantly increased the *p*AV of germ oil. The *p*AVs of samples treated at higher temperatures were considerably greater than those of stabilized at lower temperatures. The highest values were determined for the convective dry heating (160 °C), which was followed by steaming at 125 °C and by convective dry heating (90 °C).

Higher output power used for microwave treatment decreased primary oxidation products but increased secondary oxidation products. In both microwave (180 W) and dry heat treatments (90 °C), the *p*AVs were found to be much lower as a result of exposure to low temperatures. Similarly, germ samples treated by short-wave infrared radiation at 70 °C for 60 min and 80 °C for 45 min showed higher *p*AVs than raw wheat germ (*31*).

The oxidation state of the extracted oil samples was determined using the total oxidation (TOTOX) equation. The obtained results are in Table 1. The stabilized samples had lower results of TOTOX, as was the case with PVs.

### Non-enzymatic browning index of oil samples from stabilized wheat germ

In order to elucidate the passage of Maillard reaction products formed during thermal processing to the oil phase, the changes in non-enzymatic browning index (NEBI) were determined, and the results are in Table 1. Oil samples from raw and stabilized wheat germ had similar browning index values. The stabilization treatments did not significantly affect the browning index. However, Suri *et al.* (*32*) reported higher NEBI of peanut oil due to dry air roasting at 180 °C for 10 min, observable in the increase of absorbance at 420 nm from 0.04 to 0.160.

### DPPH radical scavenging activity of wheat germ oil

Wheat germ oil from the samples stabilized under low power microwave radiation showed the highest inhibition of DPPH (64.9%), even higher than the unstabilized wheat germ oil (58.2%). However, steaming (48.9%) and microwave at 360 W (54.6%) stabilization treatments significantly decreased the DPPH radical scavenging activity. This revealed that steaming under humid conditions caused the highest destruction in the compounds with radical scavenging activity. Even though the duration of steaming was 15 min, the sample was exposed to heat until the temperature reached 125 °C and then it was cooled down, which may be the reason for lower activity of the germ oil stabilized by steaming. Likewise, Bergonio *et al.* (*28*) reported that microwave treatments led to decrease in the antioxidant activity of brown rice.

Radical scavenging activity of the samples treated by convectional oven heating (90 and 160 °C) did not vary significantly between each other. These both treatments resulted in oil samples with radical scavenging activities closer to those of the untreated samples. During convective oven heating, antioxidant compounds resulting from Maillard reactions may have increased the DPPHvalues.

### Inactivation of enzymes

All treated wheat germ samples had significantly lower lipase and lipoxygenase activities than the untreated sample (Table 2). There was not significant difference in the lipase activity between the treatments; however, the results of lipoxygenase specific activity were significantly different among the treatments. The samples treated by steaming had the lowest lipoxygenase activity. Convectional oven heating (160 °C) was also one of the most effective treatments in the inactivation of lipoxygenase. Microwave radiation with a power of 360 W for 300 s followed these two treatments even with the values very close to untreated samples, microwave (180 W) and convective heat (90 °C) processes.

**Table 2:** Enzyme activity in wheat germ samples

			Enzyme activity/(U/g)					
Enzyme		Untreated	*P*(microwave)/W		*t*(dry heat)/°C (convective oven heating)		Wet heat (steaming)
			360	180	90	160	
Lipase		(0.076±0.009)^a^	(0.060±0.004)^b^	(0.060±0.004)^b^	(0.057±0.003)^b^	(0.064±0.006)^b^	(0.059±0.005)^b^
Lipoxygenase		(0.9±0.1)^a^	(0.7±0.2)^ab^	(0.8±0.2)^a^	(0.87±0.07)^a^	(0.5±0.1)^b^	(0.22±0.04)^c^

Results are expressed as mean value±standard deviation of three determinations. Different lower-case letters in the same row represent significant differences at p<0.05 (comparison between stabilized and unstabilised samples)

Hu *et al.* (*27*) reported that supersteam treatment was more efficient than hot air one for enzyme inactivation. Ling *et al.* (*33*) found radio-frequency heating to 90 °C for a few seconds did not completely inactivate lipase activity in wheat germ. Kapranchikov *et al.* (*34*) also reported that the lipase in wheat germ is heat-stable and retains over 20% of its original activitiy after being kept at 70 °C for up to 1 h.

Treatment time was found to be an important factor for the residual lipase activity as well as a linear association between the final residual enzymatic activity and air temperature (*6*). Furthermore, Xu *et al.* (*35*) reported that a combination of higher temperature and longer time was more effective in the inactivation of enzymes. Inactivation of lipase in wheat germ by microwaves was attributed to thermal effects rather than non-thermal effect, and was more effective than conventional heating in the study of Chen *et al.* (*36*).

The different secondary structure of the lipase allows the plasma to be more selective for inactivation of this enzyme. The lower rate of inactivation of lipoxygenase than of lipase inactivation can be explained by this selectivity (*12*). Lipoxygenase was less inactivated than lipase in all heat treatments, including direct steaming, drum drying, fluidized bed drying and atmospheric cold plasma treatment (*12*, *15*).

### Effects of stabilization methods on mass fractions of tocopherols and tocotrienols

Total mass fraction of tocopherols in wheat germ oil samples ranged between 960.8 and 980.9 mg/kg. All the treatments showed insignificant effect on total tocopherol mass fraction except steaming, which was the only treatment that significantly reduced total tocopherolmass fraction. Similarly, more than 96.43% original tocopherol content was reported to remain in the wheat germ samples stabilized by short-wave infrared radiation at 90 °C for 20 min (*31*). Gili *et al.* (*9*) reported total tocopherol mass fraction between 4133 and 4181 mg/kg in raw wheat germ and between 3395 and 3899 mg/kg in treated wheat germ. The values determined in the present study are lower than those reported by Gili *et al.* (*9*). The authors also observed slight effect of thermal treatments on total tocopherol mass fraction.

β-Tocopherol is the cause of the significant difference observed during steaming, as wheat germ samples stabilized by steaming lost significant amount of their original content of this homologue. On the contrary, γ- and δ-tocopherols were abundant with higher mass fractions in steamed samples than those of stabilized and raw samples. α-Tocopherol, as the predominant homologue among the tocopherols, was not significantly influenced by stabilization processes. However, tocopherol isomers in rice bran were significantly affected by infrared radiation and the most influenced isomer was α-tocopherol, as reported in the study of Yılmaz *et al.* (*37*).

As shown in Table 3, β-tocotrienol was the predominant component (54.8-77.5 mg/kg), followed by α-tocotrienol (7.6-12.5 mg/kg), γ-tocotrienol (1.1-1.9 mg/kg) and δ-tocotrienol (0.18-0.32 mg/kg). Tocotrienol isomers remained at the highest values in steam-treated samples compared to other stabilized and raw samples; the same trend was determined for γ- and δ-tocopherols as well.

**Table 3:** Mass fractions of tocopherols and tocotrienols in cold pressed wheat germ oil samples stabilized with different methods

*w*/(mg/kg)						
Isomer	Untreated	*P*(microwave)/W		*t*(dry heat)/°C (convective oven heating)		Wet heat (steaming)
		360	180	90	160	
α-tocotrienol	(8.45±0.08)^b^	(8.38±0.05)^b^	(7.64±0.04)^b^	(8.49±0.05)^b^	(8.36±0.07)^b^	(12.5±0.1)^a^
β-tocotrienol	(58.0±0.1)^b^	(57.80±0.07)^b^	(54.82±0.05)^b^	(56.63±0.08)^b^	(56.82±0.07)^b^	(77.5±0.1)^a^
γ-tocotrienol	(1.31±0.01)^ab^	(1.10±0.03)^b^	(1.10±0.03)^b^	(1.08±0.02)^b^	(1.13±0.01)^b^	(1.85±0.03)^a^
δ-tocotrienol	0.23±0.02	0.32±0.01	0.25±0.01	0.18±0.03	0.24±0.01	0.28±0.02
α-tocopherol	484.0±1.1	(486.5±1.0)	487.8±1.1	485.6±1.3	486.6±1.1	488.8±1.2
β-tocopherol	(414.5±1.1)^a^	(413.0±1.2)^a^	(415.4±1.14)^a^	(415.0±1.3)^a^	(412.7±1.1)^a^	(364.5±1.2)^b^
γ-tocopherol	(12.7±0.1)^bc^	(12.19±0.08)^cd^	(11.8±0.1)^d^	(13.2±0.1)^b^	(12.8±0.1)^bc^	(14.4±0.1)^a^
δ-tocopherol	(0.59±0.04)^b^	(0.64±0.02)^b^	(0.65±0.01)^b^	(0.69±0.02)^b^	(0.63±0.03)^b^	(0.98±0.03)^a^
Total	(979.9±1.3)^a^	(980±1.2)^a^	(979.2±1.2)^a^	(980.9±1.3)^a^	(979.5±1.2)^a^	(960.8±1.2)^b^
Results are expressed as mean value±standard deviation of three determinations. Different lower-case letters in the same row represent significant differences at p<0.05 (comparison between stabilized and unstabilised samples)						

The increase in γ- and δ-tocopherol mass fractions caused by steaming was 13.7 and 66.1%, respectively, compared to untreated ones, while mass fractions of α-, β-, γ- and δ-tocotrienols increased 48.4, 33.5, 41.2 and 21.7%, respectively.

α-Tocopherol and γ-tocotrienol were the most resistant isomers to stabilization processes, as there were not significant changes in their mass fractions after heat treatments. Li *et al.* (*5*) reported that wheat germ samples stabilized with flameless catalytic infrared treatment for 6 min lost 23.82% of their original α-tocopherol.

The amount of tocotrienols increased in steamed samples. Although this increase was observed in tocopherols too (except β-tocopherol), it was clear that there was a greater increase in the tocotrienols.

Yoshida *et al.* (*38*) concluded that tocopherols and tocotrienols showed similar mobilities within the membranes, but tocotrienols were more easily transferred between the membranes and incorporated into them than tocopherols. Steaming may increase the transfer of tocotrienols with high cell membrane mobility in the cell. Therefore, the increase in the amounts of individual tocotrienols as a result of the steaming process can be somehow explained by this phenomenon.

In the study of Yılmaz *et al.* (*37*), the loss in tocopherols induced by infrared treatment was ascribed to the high surface temperature generated during radiation. Yoshida *et al.* (*38*) reported that only 80% of tocopherols remained in soybean roasted in microwave oven for 20 min.

## CONCLUSIONS

It is well known that the oxidative stability of wheat germ is commonly improved by means of heat treatments which reduce the peroxide number and increase the antioxidant activity. However, in this study several methods and conditions were compared to determine their effect on some properties of wheat germ and its oil, with special emphasis on tocopherols and tocotrienols.

Convective heating, which is currently the most Industrially used high temperature direct heat treatment, does not offer significant advantages in terms of oxidative stability and reduction in lipase activity compared to other stabilization methods. Convective oven heating at 90 °C resulted in the highest peroxide values and at 160 °C the highest *p*-anisidine values (*p*AV) were obtained. While convective heat treatment can be preferred when the energy consumption is taken into consideration, steaming is preferable to retain product quality. The steaming process obtained more reasonable results of lipoxygenase activity, peroxide and *p*AVs. However, steaming resulted in the highest destruction of β-tocopherol, which can be associated with the lowest radical scavenging activity determined in the steam-stabilized samples.

The most effective procedure that caused notable change in tocopherols was steaming. On the other hand, tocotrienol isomers remained at the highest values in steam-treated samples compared to other stabilized and raw samples, as was the case with γ- and δ-tocopherols. α-Tocopherol and δ-tocotrienol are the most resistant isomers to stabilization, no application has caused a significant change.

Minimal processing conditions (lower temperatures and output power) can be suggested for both microwave and convective heating treatments based on reduced formation of secondary oxidation products.
